# A Comprehensive Review of Electrospun Fibers, 3D-Printed Scaffolds, and Hydrogels for Cancer Therapies

**DOI:** 10.3390/polym14235278

**Published:** 2022-12-02

**Authors:** Angelika Zaszczyńska, Beata Niemczyk-Soczynska, Paweł Sajkiewicz

**Affiliations:** Laboratory of Polymers & Biomaterials, Institute of Fundamental Technological Research, Polish Academy of Sciences, Pawińskiego 5B, 02-106 Warsaw, Poland

**Keywords:** scaffolds, hydrogels, tissue engineering, polymers, anticancer treatments, cancer therapy, regenerative medicine

## Abstract

Anticancer therapies and regenerative medicine are being developed to destroy tumor cells, as well as remodel, replace, and support injured organs and tissues. Nowadays, a suitable three-dimensional structure of the scaffold and the type of cells used are crucial for creating bio-inspired organs and tissues. The materials used in medicine are made of non-degradable and degradable biomaterials and can serve as drug carriers. Developing flexible and properly targeted drug carrier systems is crucial for tissue engineering, regenerative medicine, and novel cancer treatment strategies. This review is focused on presenting innovative biomaterials, i.e., electrospun nanofibers, 3D-printed scaffolds, and hydrogels as a novel approach for anticancer treatments which are still under development and awaiting thorough optimization.

## 1. Introduction

The human body has limited regenerative abilities. Thus, it cannot heal itself from severe damage and significant defects due to congenital abnormalities and diseases [1]. Consequently, tissue engineering strategies, including stromal cell-based therapies, are being developed. The main goal is to regenerate, remodel, replace, or support damaged organs and tissues or to induce a healing process by activating the body’s self-healing capacity [2,3].

Cancer is a serious socio-economic problem affecting people of all genders and ages [4]. In 2022, 609,360 cancer deaths in the US are predicted, while in Europe, cancer deaths are estimated at 1,269,200 [5,6]. Although it is expected that lung, stomach, colorectal, breast, uterine, prostate cancer, and leukemia mortality will decrease both in the US and Europe [4,5], there are still types of cancer, such as pancreatic cancer and glioblastoma, that lead to deaths of a large number of people and are extremely challenging for oncology [3,4,7,8,9].

Current anticancer treatments are based on surgical treatments followed by radiotherapy, chemotherapy, high-intensity focused ultrasound (HIFU), and systemic therapies, i.e., chemotherapy and hormonal therapies [10,11,12]. In some cases of anti-cancer therapies, e.g., in breast cancer therapies, three main aspects are taken under consideration, namely, treatment, regeneration of the tissue, and restoration of the physical appearance. However, their implementation usually carries many side effects [13,14]. For instance, chemotherapy causes the nonspecific distribution of chemotherapeutics, resulting in decreased anticancer effects as well as systemic toxicity [15]. In addition, long-term anticancer drug intake very often leads to tumor resistance, which makes it difficult to cure cancer even with alternative treatment methods [16].

A relatively novel, promising, and still developing approach is near-infrared light (NIR)-induced photothermal therapy (PTT) as a local, minimally invasive anticancer therapy that destroys cancer cells via hyperthermia [17,18]. The PTT method is based on controlling NIR and using appropriate photothermal conversion agents that prevent damage to surrounding tissues [19,20].

**Figure 1:**
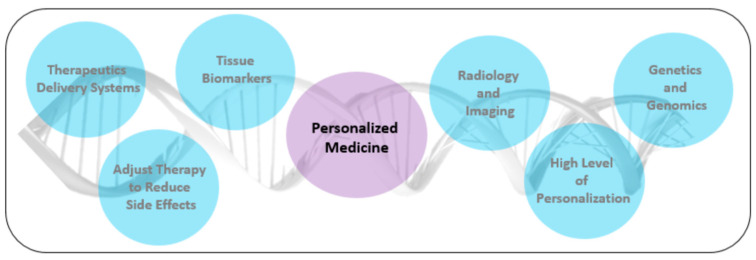
Scheme of personalized medical treatment.

Moreover, an innovative method of treatment used in the prevention, diagnostics, and cancer therapy is personalized medicine (PM, Figure 1) [21]. PM selects a specifically matched treatment based on the patient’s genome, lifestyle, and medical test results. In the case of cancer, tumor heterogeneity significantly decreases the efficacy of conventional treatments, thus requiring therapies with a higher level of personalization. Additionally, PM can adjust therapy to reduce side effects and provide efficient therapeutic delivery [13]. Implementation of such treatment requires using technologies that allow the designing and formation of biomaterials providing effective therapeutics delivery systems or tissue regeneration. In this respect, the use of various materials in regenerative medicine has been expanding rapidly in the last few years [22]. The evolution of modern biomaterials would be impossible without the special parameters of certain materials. Originally, these materials were used in tissue engineering to improve the living standards of patients. Personalized medicine can help increase the effectiveness of the cancer treatment process because the methods and active substances used in it are aimed at learning about the abnormal mechanisms underlying the disease, and then finding the right drug. Personalized medicine has many advantages because it entails the greater effectiveness of individually selected drugs, the ability to prevent the development of the disease and cure it, knowledge about the patient’s health condition, the predisposition of his body, and fewer side effects. These advantages are very important from the perspective of a patient with a weakened organism [23].

Human tissues have different biological, physical, and biochemical properties. To achieve those requirements, the choice of appropriate material is essential for effective treatment. Degradation kinetics, design aspects, and cell-material interaction are crucial in developing biomaterials. The U.S. Food and Drug Administration (FDA) has approved many materials and technologies for medical purposes, such as scaffolds, drug-delivery treatments, fabrication techniques, and implants. This multidisciplinary area’s rapid progress also allows for improving modern medical applications [24].

**Figure 2:**
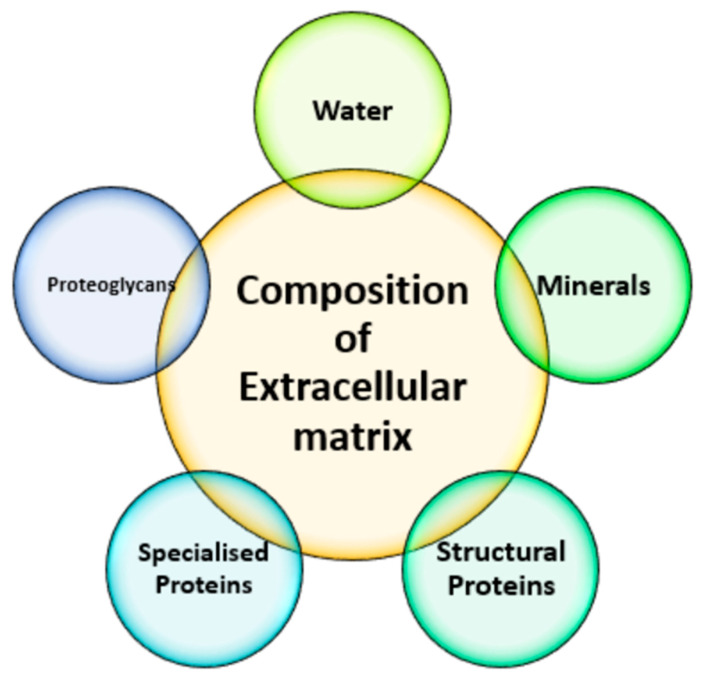
Composition of the ECM.

Ideal biomaterials must create conditions similar to those occurring in the natural extracellular matrix (ECM), that provide adequate cell adhesion, proliferation, and differentiation. An ECM is a dynamic structure that occurs between neighboring cells, which is made from water, structural proteins, minerals, proteoglycans, and specialized proteins (Figure 2) [25]. The knowledge of the composition of various tissues, ECMs, and tumor physiology plays a crucial role in understanding their function–structure relationship, and thus in anticancer and regenerative medicine [26,27].

Currently, for anticancer treatments and subsequent regenerative medicine, various polymeric materials are used due to their favorable structure, chemical composition, and molecular weight. They can be generally categorized as naturally occurring or synthetic polymers. The most important natural materials used as cell scaffolds include proteins (collagen and gelatin, fibrin, fibrinogen, silk, etc.), polysaccharides (cellulose, chitosan, dextran, hyaluronic acid, agarose, etc.), and polynucleotides (RNA, DNA). Synthetic polymers are being widely investigated, with their advantages and disadvantages compared to natural polymers. In this respect, poly (methyl methacrylate) (PMMA) [28], polycaprolactone (PCL) [29], poly (L-lactide) (PLLA) [30], poly (lactic-co-glycolic acid) (PLGA), polyvinylidene difluoride (PVDF) [31], polyglycolic acid (PGA), polyhydroxyalkanoates (PHA), poly (3-hydroxybutyrate-co-3-hydroxyvalerate) PHBV [32], poly (hydroxybutyrate) (PHB) [27], etc. have been distinguished. In addition, many scientists test a combination of polymers and additives to obtain better biological and mechanical properties [33].

The main goal of this paper was to present a review of the published research concerning materials for anticancer therapies and tissue regeneration after resection. Current approaches to biomaterials, i.e., electrospun nanofibers, 3D-printed scaffolds, and hydrogels in the field of modern medicine are reported. We further discuss the novelties in the field of new technologies, cell, and drug delivery systems, and their use as approaches for cancer therapies.

## 2. Fibrous Scaffolds for Anticancer Treatments

Progress in materials science and human expectations have caused the development of biodegradable and non-biodegradable scaffolds for restoring the function of damaged tissues [34]. Some of them have found applications in cancer therapy. Materials and methods for forming highly functional scaffolds are discussed below.

There exist several types of nanofibers scaffolds manufacturing dedicated to tissue engineering and anticancer treatment. The most widely investigated is the electrospinning process, but there are also other methods, such as bicomponent fiber spinning, solution blowing, melt blowing, phase separation, drawing, and others [35].

**Figure 3:**
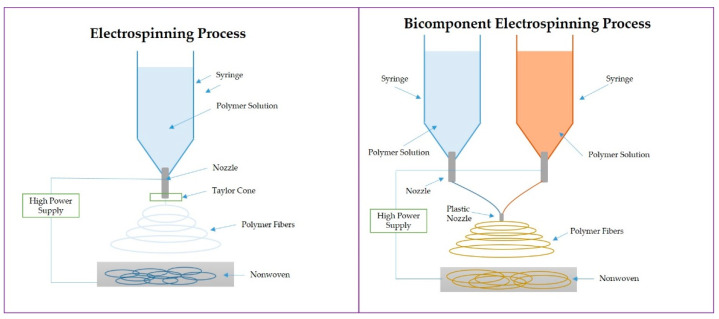
Scheme of the electrospinning process (**left**) and bicomponent electrospinning process (**right**).

Electrospinning (Figure 3.) is a method of manufacturing nanofibers with the use of a high electric voltage. In the electrospinning technique, a strong electric field is applied to the liquid polymer (or melt-melt electrospinning), resulting in the distribution of electric charges on the surface of the polymer droplet coming from the needle. An electrode with positive potential is connected to the capillary with the polymer solution, and another, with negative potential, to the collector. It is worth noting that manufactured nanofiber mats have a high surface area and controllable pores and can potentially diagnose and treat cancer cells [36]. Nanofibers, with the addition of anticancer drugs, can give sustained release after cancer tumor removal [37]; however, there is a need to produce more advanced materials which will support the complicated treatment after tumor removal [38].

Thus, based on the electrospinning technique, various methods of complex nanofiber production have been developed. Bicomponent spinning (BCS, Figure 3 (right)) allows the manufacturing of nanofibers in a two-step process. Two polymers can be produced by splitting or removing components [39] and, with the dual release, can be used in anticancer therapy [40]. Another type, coaxial electrospinning (CEs), allows the production of core-shell fibers and offers an advanced system of drug carrier options for multiple drug delivery [41]. Multi-jet electrospinning (MNEs) allows the production of nanofibers using several jets, which improves productivity [42], although it can result in poor fiber quality. Emulsion electrospinning (EEs) produces fibers from two immiscible solutions; nevertheless, due to the high number of variables, the fibers are difficult to produce [43].

**Figure 4:**
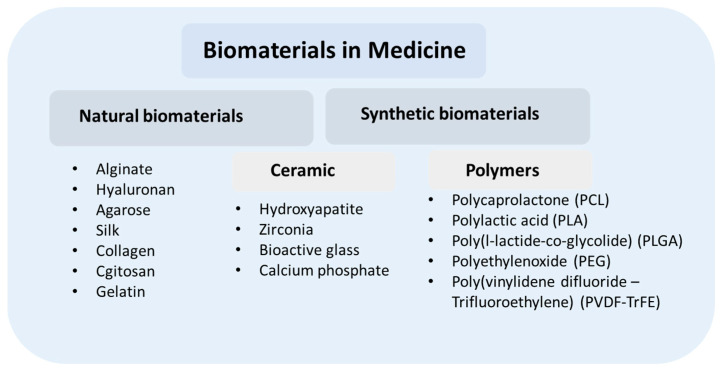
Classification of materials used in medicine.

Aside from electrospinning methods, several non-electrospinning techniques have been developed for improving nanofiber production; these include phase separation, solution blowing, template synthesis, freeze/drying synthesis, interfacial polymerization, drawing techniques, and splitting [44]. These techniques use centrifugal force or gases instead of electricity to produce nanofibers, but they yield low-quality fibers, and thus their application in tissue engineering is limited [45]. Depending on the type of process and parameters, different types and shapes of materials can be manufactured, such as nanowires, nano-webs, porous fibers, random and aligned nanofibers, ribbons, and others [46]. Figure 4 presents the classification and examples of materials used in medicine.

### 2.1. Degradable Scaffolds

Degradable polymers, the most preferable candidates as scaffolds for tissue engineering, can be further classified in terms of their origin as natural or synthetic. In anticancer treatments, degradable polymers, e.g., poly (vinyl alcohol) (PVA), poly (β-hydroxybutyrate-β-hydroxyvalerate) (PHBV), poly (lactic-co-glycolic acid) (PLGA), polycaprolactone (PCL), and polylactic acid (PLA) nanofibers, serve as scaffolds and site-specific drug delivery systems [47,48,49,50].

#### 2.1.1. Natural Degradable Polymers

Natural degradable polymers are incessantly investigated in anticancer treatment. Nowadays, 3D silk is gaining interest, because 3D silk scaffolds are biodegradable and easy to form, and have excellent mechanical properties [51]. Electrospinning gives the opportunity to obtain very thin silk fibers (SF) with a diameter of <500 nm [52]. Recently, to develop scaffolds with good mechanical properties, scientists used SF for reinforcing hydrogels [53]. SF and silk-sericin (SS) are perfect materials for use as a biotemplate to develop lung anticancer system drug delivery systems [54]. They are widely used as chemotherapeutics delivery systems. For instance, SF/PCL fibers have been used to release titanocene, a drug for breast cancer therapies resulting in the promotion of MCF-7 breast cancer cell apoptosis [55]. Especially important here were interactions between SF amine groups and titanocene, which increased the rate of cancer cell apoptosis. In another study reported by Li et al. [56], silk regenerated SF loaded with curcumin (CUR, hydrophobic drug) and doxorubicin hydrochloride (DOX·HCl, hydrophilic drug) were colloid-electrospun to provide the dual drug in a sustained way for breast and skin anticancer therapies. TEM microscopy has confirmed the effectiveness of the electrospinning by the presence of both drugs. Additionally, both drugs showed controlled release according to the Fickian model. Such an approach was a preliminary study, and, in the future, it should be tested on breast or skin tumors to evaluate its usefulness for anticancer treatments.

Another natural material, collagen, can serve as an excellent scaffold for supporting cell–matrix interactions and cell proliferation, adhesion, and migration [57]. To achieve high mechanical strength, collagen can be mixed with synthetic polymers such as PGA, PLA, or P (LLA-CL) [58,59]. Collagen has a high degradation rate, biocompatibility, and minimum immune response [60]. Collagen scaffolds are widely investigated in medicine, including for wound healing [61], tissue engineering, drug delivery systems [62], and other tissue engineering applications [63]. Many experiments have shown the influence of specific collagen on anticancer treatments. Typical matrix collagen COLI takes place in ECM remodeling, which in effect induces metastasis or invasion of epithelial ovarian cancer cells; after remodeling, it affects the ovarian cancer cells by signaling pathways [64]. These studies have shown that ECM remodeling increases the invasion of aggressive ovarian cells by adequate signaling and the further blocking of these signaling pathways could result in the successful destruction of ovarian cell proliferation and further invasion.

In other studies, PCL/Collagen and piperine served as anti-breast cancer drug delivery systems. Based on the results, such combinations showed good mechanical properties, influenced sustained piperine release, decreased tumor size, and induced apoptosis in MCF-7 and 4T1 breast tumor cell lines. On the one hand, collagen is a material that brings many advantages, but on the other, collagen is an expensive material [52], and there are plenty of polymers that are cheaper alternatives showing comparable properties. One example is gelatin. Gelatin has found many applications in industry as well as in biomedicine. Further, after chemical modification or blending with different polymers it can be used as a biomaterial in long-term applications, such as brain hydrogel [65]. The anticancer use of gelatin in modern anticancer therapies additionally provides PTT effect. Such a combination is described in detail below, in Section 2.2.

Another natural material, alginate, can be used as a biomedical scaffold due to its viscoelastic properties [66]. Alginate shows good biocompatibility, nontoxicity, and low price, and can be crosslinked with Ca^2+^ ions. Chen et al. [67] formed an interesting composite consisting of freeze-dried alginate/gelatin sponge and curcumin-loaded electrospun fibers (CFAGS) for wound healing after tumor resection. The results showed that CFAGS released curcumin stably during the entire study (15 days). In vitro and in vivo studies showed that stable curcumin release destroyed MCF-7 tumor cells and prevented tumor recurrence after surgery.

Chitosan is a biomaterial with high solubility properties and cationic nature [68]. It can be applied to organs, nerves, and cardiac tissue due to its similarity to the extracellular matrix (ECM) [69]. Chitosan combinations with poly (vinyl pyrrolidone) (PVP) [70], poly (vinyl alcohol) (PVA) [71], or natural materials gelatin and collagen are usually formed via electrospinning as anticancer drug delivery systems. Similarly to SF, the free amine groups that are present in chitosan can interact with hydrophilic anticancer drugs. This feature makes it favorable from the anticancer drug delivery perspective. Shafabakhsh et al. [72] described that the interactions between chitosan and anticancer drugs resulted in an effective decrease in tumor cell proliferation, higher apoptosis, as well as decreased metastasis.

#### 2.1.2. Synthetic Degradable Polymers

The most common synthetic degradable polymers dedicated to anticancer therapies are listed below.

PLA is widely used in resorbable medical sutures due to its non-toxic nature [73]. In tissue engineering, various materials are mixed with PLA polymer to achieve higher mechanical properties. In bone tissue engineering, PLA-based composites, such as PLA-hydroxyapatite, PLA-chitosan, PLA-PLDA, and PLA-phosphates have been created [74,75]. To achieve a composite with high stiffness, PLA with tricalcium phosphate was mixed and applied as a bone implant [76]. PLA biomaterials can be manufactured using different technologies, such as film casting, thermoforming, electrospinning, and nano- and micro-methods, and can be formed into various sizes and shapes to be used as a medical scaffold [77].

In oncology, PLA-based scaffolds can be used in drug delivery due to the incorporation of chemotherapeutic agents inside fibers [78]. For instance, Yuan et al. [79] formed doxorubicin (DOX)-loaded mesoporous silica nano-particle with PLLA nanofibers composite showing early phase and sustained drug release for breast cancer therapies. The results showed successful sustained DOX release from 50 to 120 days, which is extremely long in comparison to other drug delivery systems. Additionally, the composite induced MDA-MB-231 breast tumor cell with decreased Bcl-2 and TNF-α gene expression. Considering controlled prolonged drug release and positive anticancer response, such fibrous composite has huge potential for future anticancer treatments.

Another example of a synthetic polymer is PLGA. The degradation of PLGA can result in an acidic environment. Tumor environments are also usually acidic, so PLGA could be useful or not depending on its precise application [80]. PLGA is widely examined in drug delivery systems [81] and as a nanoparticle-based system in cancer tumor treatment [82]. An interesting approach is a PLGA electrospun mat loaded with Ag nanoparticles [83] for liver anticancer therapies. In vitro studies on Hep-G2 cell lines showed the anticancer character of PLGA fibers and the anticancer effect increased with the Ag nanoparticles contribution in the fibers. In other studies, PLGA [84] nanofibers were loaded with metformin to study the anticancer effect on A549 human lung adenocarcinoma cells. The results showed sustained drug release over two weeks and subsequent polymer degradation after ca. 24 days. The controlled release of metformin resulted in a cytotoxic effect on A549 tumor cells after two days, which resulted in their apoptosis.

PCL is another polymer commonly used in electrospinning for fibrous mat formation dedicated to anticancer therapies. PCL is one of the most promising biodegradable biomaterials approved by the Food and Drug Administration (FDA) as sutures, drug delivery devices, and adhesion barriers. In cancer treatment, nanoparticulated PCL-based drug delivery systems have much to offer [85]. Additional advantages of these drug systems include drug transport to the injured tissue, protection of the drug from disintegration, and stability in biological fluids compared to conventional chemotherapy [86]. Various drug delivery systems based on PCL have been investigated in glioblastoma, a primary brain tumor. For instance, [87] core-shell electrospun PCL/mycophenolic acid (MPA) was studied by Han et al. The results showed an effective anticancer effect of such an approach through hampering glioblastoma U-87 MG multiforme cell growth. The drug delivery system provided sustained drug release after 100 h. The authors understand the need to release the drugs over a longer time (ca. 49 days) and characterized the polymers more to provide longer drug release to fulfill anticancer treatment needs. In other studies, [88] Irani et al. formed PCL-Diol-b-PU/Au/temozolomide (TMZ) nanofibers for glioma tumor treatments. On the one hand, the results showed a cytotoxic effect of the composite against U-87 human glioblastoma, confirming the chemical composition is adequate for further, more advanced anti-glioblastoma therapy studies. On the other hand, however, the study showed drug release for 24 h only, which is too short for the actual needs of anti-cancer therapies. The prolonged TMZ release studies should be carried out in the future or the chemical composition of such an approach should be adjusted more thoroughly.

PVA is another polymer widely used in tissue engineering and anticancer therapies [89]. To achieve desired properties, such as high mechanical strength, PVA, similarly to other polymers, could be blended with various types of materials. For instance, PCL/PVA core-shell electrospun fibers [90] were loaded with the anticancer drug paclitaxel (PTX). PCL was a core, while PVA served as a shell, and combining both of these polymers led to obtaining a biocompatible and biodegradable composite, while PTX is an anticancer drug that additionally shows pH sensitivity. The drug delivery system showed the sustained release of PTX under acidic pH after over 25 days. This indicates that PCL/PVA/PTX could be useful in anticancer drug delivery. Additionally, the composite, due to its sustained drug release, inhibited the proliferation and growth of LoVo colon cancer cells, resulting in their destruction. Those results indicated that such an approach has great application potential in the chemotherapy of some solid tumors in the clinical setting. An overview of electrospun degradable and non-degradable polymers and their biomedical applications is given in Table 1.

### 2.2. Electrospun Scaffolds Combined with PTT Effect

An interesting approach that is currently studied is broad of elecrospun fibers functionalities via for instance conjugation of electrospun mats with PTT effect. This conjugation involves both natural and synthetic degradable polymers.

A great representative of natural polymers is gelatin. There are many scientific reports describing its positive effect with PTT for anticancer treatments. For instance, a gelatin scaffold with gold nanoparticles can be used in photothermal cancer therapy [65]. Zhang et al. [65] adopted such an approach, which besides good biocompatibility, upon NIR laser irradiation at 805 nm heated up to 50–60 °C. The photothermal effect resulted in the effective destruction of HeLa cancer cells. A similar study used a folic acid/gelatin/gold nanoparticles composite scaffold for the photothermal ablation of breast cancer cells [66]. The gold nanoparticles generated a photothermal effect under an NIR laser of 805 nm, while folic acid served as a targeting ligand in nanoparticles. Additionally, folic acid could recognize and bind the specific receptors overexpressed by breast cancer cells. The results showed the material heated up under an NIR laser up to 35.1 °C, which resulted in the local destruction of MDA-MB231 breast cancer cells.

The incorporation of the PTT effect with synthetic electrospun fibers is currently being studied as well. For instance, Cheng et al. [108] formed gold nanorods (GNR) polyethylenoxide (PEG) and PLGA composite fibers which served as membranes as a photothermal platform for anticancer therapy. Not only did such an approach show an excellent photothermal effect resulting in effective tumor cell destruction but also the biodegradability of the mat provided tissue regeneration after resection. The presence of GNRs allowed the material to produce heat up to 42 °C at NIR light of 850 nm, which effectively destroyed HeLa and breast cancer MCF-7 cells.

Another trend in anticancer approach designing is the combination of the PTT effect and chemotherapy. Such a combination allows adequate anticancer drug delivery via electro-spun mats and at the same time provides additional therapy that will strengthen cancer cell destruction. Chen et al. [109] combined electrospun fibers to release in a controlled way chemotherapeutics with PTT effect to effectively destroy cancer stem cells (CSCs). In this respect, PCL nanofibers released all-trans retinoic acid (ATRA) drugs, while multi-walled carbon nanotubes (MWCNTs-OH) served as PTT agents. The ATRA and MWCNTs-OH were placed in PCL solution and electrospun. The obtained composite fibers were capable of sustaining drug release for a long time (more than 15 days). The presence of MWCNTs-OH increased the mechanical properties of the composite materials and created heat in the range of 42–47 °C under NIR light (808 nm) to destroy CSCs. Additionally, ATRA stimulated CSC differentiation improving the PTT effect. Treatment with composite fibers with the PTT effect showed a decrease in the mice’s tumor size.

Another interesting approach was reported by Azerbaijan et al. [110], where pH-sensitive core-shell nanofibers with PTT effect were produced of molecules doped with poly (tetramethylene ether) glycol-based polyurethane (PTMG-PU) which served as a core, paclitaxel, graphene oxide/gold (GO/Au) nanorods loaded into PTMG-PU, and chitosan. Chitosan served as a shell. In this composite, paclitaxel was chemotherapeutic and the GO/Au nanorods were PTT agents while chitosan performed pH sensitivity promoting the acceleration of anticancer drug release in an acidic (cancer) environment.

The composite showed an excellent PTT effect, where the material heated up to 51–55 °C under 808 nm laser irradiation. Additionally, chemotherapeutic was released in a controllable manner near the tumor area due to the PTT effect and pH sensitivity. The results showed an increase in apoptotic nuclei in A549 lung cancer cell lines indicating the huge potential of such an approach for future anticancer treatments. Other examples of electrospun scaffolds combined with PTT effect were shown in Table 2.

## 3. 3D Printed Scaffolds and Hydrogels for Anticancer Treatments

**Figure 5:**
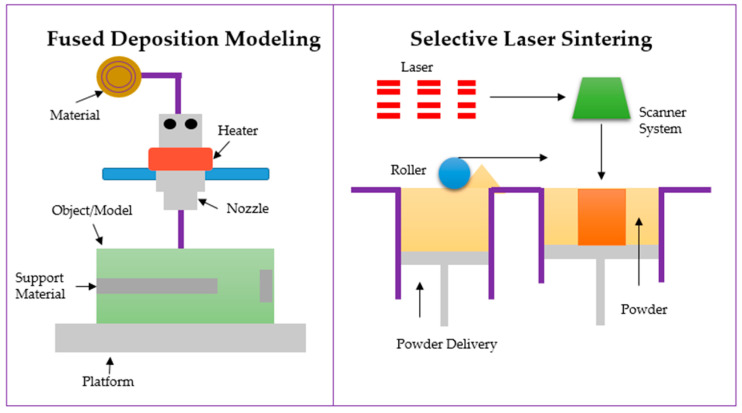
Scheme of the FDM (**left**) and SLS (**right**) process.

A 3D-printing method allows many types of scaffold manufacturing. The 3D printing might be classified in terms of the techniques used e.g., fused deposition modeling (FDM, Figure 5, left), inkjet printing, laser beam melting, selective laser sintering (SLS, Figure 5, right), bioprinting, extrusion, digital laser printing (DLP), polyjet, stereolithography, and electron beam melting [118,119,120]. Additionally, the method could be divided into classical printing [121] and bioink, i.e., 3D printing with cells or bioactive substances [122,123].

The 3D printers currently in use are based on three principles: liquid solidification, powder solidification, and extrusion. The most popular method of 3D printing is SLA. This method uses the phenomenon of photopolymerization, which causes the solidification of the liquid. The process is repeated layer by layer until an object is created. In the SLS method, the laser heats and melts the powder, which creates a 3D object. The first step is to distribute the powder on the platform evenly, and then the roller aligns the surface of the object. Another method of inkjet 3D printing is to print objects drop by drop. The droplets sprayed from the nozzle are applied in thin layers and cured with high-energy light or cool air. FDM is a method based on the extrusion of material from a nozzle and distribution in layers on the worktable. Each method has its pros and cons, which are detailed in Table 3. This does not change the fact that 3D printing is a promising tool in personalized medicine, which tries to treat cancer patients [124].

Like all scaffold manufacturing methods, this one also has its pros and cons. On the one hand, the 3D printing method brings great advantages, i.e., easily accessible, easy processing, variety of bioprinter types, relatively low costs, generates less waste, and most importantly, the ability to form precisely controlled structures [129,130]. On the other, depending on the chosen material, bioprinter, and fabrication process still, there are some problems with the restricted built size, not-fully crosslinked material, or cell aggregation that leads to the nozzle tip clogging [131].

Since studies of anticancer therapies involve a wide spectrum of diagnosis, treatments, prognosis, and metastasis, 3D printing is a technique that could play a role in all these areas. The 3D-printed materials might be applied as tumor models (3D cell culture models), personalized drug delivery systems, bioprinted organs, organ-on-chip models, and devices used in diagnosis [132]. Three-dimensional-bioprinted scaffolds, including aliphatic polyester or hydrogels for cancer therapies, are also formed mainly for treating residual cancer after the resection of laminin-functionalized PDLLA surgery but also serve as a 3D cell culture model [133,134].

Currently, there is a clear trend of using 3D-printed materials that provide the synergistic effects of using current anti-cancer therapies (chemotherapy, radiotherapy, PTT, and others), promoting tissue regeneration after resection, and avoiding cancer relapse. On the one hand, such approaches increase the scope of their applications and face most of the requirements imposed by cancer therapies; on the other, the fabrication of such biomaterial is challenging and complex, and requires laborious and multi-step functionalization [135]. Moreover, functionalization could change the scaffold properties such as biodegradation or mechanical properties.

### 3.1. Three-Dimensional-Printed Scaffolds Combined with PTT Effect

Three-dimensional-printed scaffolds provide many benefits from the tissue engineering point of view. One of them is interconnected pores and controlled porosity, which could provide a decent environment for cellular activities as well as nutrient and gas transportation [136]. Another is customized parameters of the scaffolds, i.e., architecture, composition, and stiffness. However, for anticancer treatment providing all of the above-mentioned parameters is still insufficient. Combining 3D printing as a scaffold preparation method triggers the ablation of tumors through hyperthermia and the simultaneous promotion of tissue regeneration [137]. PTT could promote immunoadjuvant-like effects to generate the immunity responsible for tumor-attacking [138]. The photothermal agents are usually metals, metal oxides, NIR dyes, graphenes, and others [139,140]. The photothermal surfaces are fabricated via firm immobilization or deposition of photothermal agents or via direct fabrication of materials showing the photothermal effect [141]. The greatest advantage of photothermal material is providing precise control of time, heat area, and intensity by adjusting light irradiation. The wavelength that activates the photothermal effect is in the range of 700–1400 nm [142]. By combining such approaches with ceramics or polymers, novel multifunctional scaffolds are formed and presented in Table 2 [143,144,145].

Most of the current activity is focused on the 3D printing of ceramic scaffolds providing the PTT effect. For instance, He et al. [146] formed a thermoactive biodegradable 3D-printed scaffold consisting of immune adjuvant (R837)-loaded and niobium carbide (Nb_2_C) MXene-modified bioglass (BG@NbSiR) as bone metastasis of breast cancer therapy. This strategy was based on the PTT and immune-activation properties of NS Nb_2_C@Si loaded with R837. This multifunctional scaffold stimulated long-term immune memory, providing adequate protection against breast cancer metastasis. Additionally, the unique properties of the material and the biodegradation of BG@NbSiR provided more efficient bone regeneration. Similar studies were conducted by Ma et al. [147], who designed the Fe-CaSiO_3_ scaffold using a facile ball-milling and 3D printing technique as a bone cancer treatment. The scaffolds combined high compressive strength, photothermal effect, and reactive oxygen species (ROS) production that is harmful to tumor cells, and provided effective bone regeneration. The high compressive strength (up to 126 MPa) allowed it to fill, withstand, and mechanically support bone cortical defects. It is reported that the compressive modulus of human bone is in the range of 90–170  MPa. Additionally, such scaffolds provide excellent photothermal effects, anticancer therapeutic effects as a result of Fe ions, and sustained release. Fe ions catalyze the degradation of H_2_O_2_ in tumor cells resulting in ROS production. In vitro studies have shown improved rat bone mesenchymal stem cells (rBMSCs) adhesion and their osteogenic differentiation, while in vivo tests on New Zealand rabbits confirmed the scaffold’s bone regenerative effect.

An interesting approach is the development of polymer/ceramic-based scaffolds for bone cancer treatment. One example is an approach developed by Yang et al. [140]. The 3D-printed SrCuSi_4_O_10_/PCL scaffold was developed for inducing osteosarcoma ablation and providing effective bone vascularization. The composite scaffold provided an excellent PTT effect for osteosarcoma without side effects. Additionally, sustained Sr, Si, and Cu ions release increased rBMSCs adhesion, proliferation, and osteogenic differentiation. In other studies, such as that by Wang et al. [145], the 3D-printed borosilicate bioactive glass (BG) was functionalized with MoS_2_-PLGA film to provide a PTT effect. Such composite not only decreased the viability of MNNG/HOS osteosarcoma cells in vitro and stopped tumor development in mice in vivo but also stimulated the proliferation and osteogenic differentiation of rBMSCs. The 3D-printed polymeric/ceramic multifunctional scaffolds with the PTT effect are still relatively new approaches, but the authors believe that the development of this particular field of materials science will provide many benefits in future anticancer treatments. The 3D-printed scaffolds combined with the PTT effect are summarized in Table 4.

#### Three-Dimensional-Printed Hydrogels Combined with PTT Effect

Hydrogels are highly hydrated, three-dimensional polymers that mimic native ECM and, at the same time, might serve as drug/cell/growth factor delivery systems and scaffolds. The tunable mechanical and biochemical properties are another advantage of these materials [153]. Similar to 3D-printed scaffolds, due to the complex and dynamic tumor microenvironment, anticancer approaches based on hydrogels must be multifunctional and targeted. They should provide more features than a conventional hydrogel scaffold, e.g., the PTT effect [154]. One such hydrogel-based therapy is gelatin (Gel), sodium alginate (Alg) hydrogel system loaded with CuO nanoparticles obtained by Dang et al. [155]. This approach combined the photothermal effect and provided biochemical cues resulting in tumor recurrence. Gel and Alg provided good biocompatibility, biodegradability, controlled release of CuO nanoparticles, and 3D printability, while CuO nanoparticles served as photothermal agents and released Cu ions in a controllable way, resulting in ROS production.

In other studies, conducted by Lia et al. [133], methacrylated gelatin (GelMA) and methacrylated chondroitin sulfate (CSMA) hydrogels were loaded with gold nanorods (GNRs) and nanohydroxyapatite (nHA) for bone cancer treatments. Hydrogel components provided biocompatibility and 3D printability, the GNRs provided a PTT effect at 808 nm, while nHA mimicked the native bone ECM, and promoted osteogenic differentiation of MSCs and bone mineralization, which led to bone regeneration in defect areas previously invaded by a tumor.

The conjugation of scaffolding, local chemotherapy, and the PTT effect is also an interesting therapeutic direction. Xu et al. [134] designed 3D-printed sodium alginate (SA), gellan gum (GG), and polydopamine nanoparticles (PDA NPs) loaded with the chemotherapeutic of doxorubicin (DOX). In this composite, SA-GG provided biocompatibility, thermal sensitivity, and 3D printability, while PDA NPs provided a great PTT effect at 808 nm, ca. 1.5-fold increased mechanical properties of the scaffold, and provided increased wound healing ability after surgery. Combining PTT and chemotherapy provided significantly lower viability of B_16_F_10_ tumor cells and prevented tumor recurrence.

In recent times, a significant trend observed during scaffold design has been to conjugate current anti-cancer therapies and diagnostic methods. Such a smart scaffold could provide tissue reconstruction after breast cancer resection, photothermal conversion required for PTT, as well as a scaffold imaging possibility via photoacoustic imaging (PAI) or magnetic resonance imaging (MRI) [135]. In this regard, Luo et al. designed 3D-printed dopamine (DA)-modified sodium alginate/polydopamine (Alg-PDA) scaffold for breast cancer treatment. In this system, Alg provided desirable biocompatibility and safe crosslinking using Mn^2+^ cations. Not only did Mn^2+^ play a crucial role as a crosslinking agent and chemically coordinated PDA but also served as a contrast agent used in MRI. While DA and PDA increased cellular response due to the presence of catechol moieties, provided a photothermal effect for PTT, and allowed diagnosis via PAI and MRI. Such a 3D scaffold was characterized by increased porosity, with the pore size ranging from 1 μm to 1 mm, which provided adequate vascularization and cell infiltration. Additionally, the Alg-PDA scaffold showed mechanical properties of ca. 2 kPa, which were comparable to the human breast tissue (ca. 3 kPa), and an excellent photothermal effect of 2-fold increased temperature after MRC laser exposure. Most of all, multiple exposures by laser did not weaken the material mechanically. Conjugating the scaffolding with laser exposure showed over 60% decreased viability of 4T1 breast cancer cells. Simultaneously, the scaffolds provided good proliferation of MCF-10A breast cells showing good potential for anti-cancer treatments and further tissue reconstruction.

### 3.2. Hydrogels as 3D Cell Culture Models for Cancer Therapies

Cell culture models are biomedical approaches that monitor tumor cell behavior and signaling pathways, which is important information from the perspective of anti-cancer treatments. Currently, two types of cell culture models can be distinguished, 2D and 3D. The 2D cell culture models are simple and inexpensive [149]. However, they do not reflect complex 3D tumor environments [9]. The cell-cell and cell-ECM interactions strongly influence cell behavior. In 2D models, those interactions are damped, which most likely leads to cell dysfunction, and this has been observed in many cell types, such as hepatocytes or colorectal cancer cells [149,150,151]. Additionally, cancer cells seeded on 2D models do not show the real drug resistance observed in in vivo conditions.

A promising alternative that overcomes those limitations is the 3D cell culture model. The 3D cell culture models are more biochemically diverse and structurally complex than 2D models, enabling cell-cell and cell-ECM interactions and providing the same environmental conditions as in vivo [9,149]. Cells growing in 3D scaffolds show a slower proliferation rate and restore the histological differentiation characteristic of primary tumors that were not present in 2D cultures. Cells seeded on various 3D models also show varying cell morphology, various gene expressions, as well as various levels of drug resistance [9,151,156,157,158,159]. Ruedinger et al. reported cells seeded on 3D models to show a slower proliferation rate and present histological differentiation characteristics for primary tumors. Such a phenomenon is not observed for 2D cell culture models [160]. Hence, many types of 3D culture models are currently available. In this instance, spheroids [161], organoids [162], microfluidic devices [163], microfibers [164], and hydrogels [165] can be distinguished.

Hydrogels dedicated to cancer therapies very often serve as biomedical 3D approaches which accurately reflect the tumor microenvironments. To fairly mimic the native tumor microenvironment, such hydrogels should correspond mechanically and physiologically to the tumor but also to native healthy tissue [166]. As with scaffolds for tissue engineering, cells are grown on 3D scaffolds in in vitro conditions that induce adequate regulation of specific gene markers of tumor cells. In this section, hydrogels as an attractive approach applied to 3D cell culture models will be discussed. Hydrogels are especially interesting candidates for monitoring soft tissue cancers, e.g., glioma or glioblastoma, and breast cancer cells. Hydrogels well mimic the aqueous environment of human tissues, and their biological and mechanical properties, porosity, gasses, and nutrient diffusion can be easily adjusted.

#### 3.2.1. Natural Hydrogels

Since cancer cells mostly respond to the biochemical and mechanical cues of the tissue, a huge impact is imposed on adequate adjustment of those properties during 3D model designing. Additionally culturing of tumor cells within 3D allows for acquiring phenotypes and response to stimuli that are observed in vivo conditions. [167]. Thus, cancer drug delivery systems can be optimized using 3D platforms like hydrogels [168]. Due to the ability to mimic key characteristics of in vivo tumor progression, there are many examples of hydrogels that serve as 3D models for cancer therapies. The most common natural hydrogels used as 3D models are mostly proteins and polysaccharides [169,170].

Collagen I is a protein hydrogel that shows great biocompatibility and could easily mimic the native environment of a tumor. The most important feature of this hydrogel is the presence of tripeptide RGD (Arg-Gly-Asp) which effectively binds the cell surface receptors [167]. Additionally, Collagen I promotes glioma cell growth, forming spheroid aggregates, and allows us to understand the tumor’s properties such as growth, proliferation, and invasion [171]. In studies conducted by Szot et al., the 3D collagen I cell culture model provided fast growth of MDA-MB-231 breast cancer cells with the parallel capability of necrotic and hypoxic areas development. Additionally, cell-cell and cell-ECM interactions resulted in cell signaling and their phenotype observed in 3D models reflected in vivo conditions reality. Despite simplification of the tumor’s complex nature, these studies allowed the study and reproduction of tumor necrosis, hypoxia, or gene expression that natively occur in vivo conditions.

In other studies, Jia et al. [172] studied the changes and key genes and miRNA affecting stemness functions and anticancer drug sensitivity of U87, U251, and HS683 glioma cells cultured in 3D collagen I culture model. Collagen I enabled glioma colony formation and evaluated their drug resistance. Studies on 3D models have allowed the investigation of 77 genes of glioma as well as signaling paths of protein network interactions as a response to stresses, DNA damage/repair, and drug metabolism. In these studies, it was discovered that AKT1, ATM, CASP3, CCND1, EGFR, PARP1, and TP53 genes and miRNA in glioma cells showed related pathways increasing the stemness and decreasing drug sensitivity of glioma, which suggests their crucial role in future diagnosis and further potential treatments.

Hyaluronans, i.e., hyaluronic acid or its salts, are polysaccharides that represent natural hydrogels [173]. The molecules of HA natively occur in native ECM, and HA has easily tunable mechanical properties through adjustment of concentration and crosslinking degree. The great advantage of HA is the ability to modulate healthy and tumor cells’ fate [174].

Additionally, HA content, especially synthase gene expression in HA, is upregulated in many cancers, such as breast, pancreatic, colon, lung, and prostate cancer, and thus is a perfect material for 3D tumor cell culturing [175,176,177,178,179]. It is reported that a high content of synthase gene expression correlates with higher mortality in patients. Thus, the investigation of tumor cells on HA-based 3D platforms is fully justified. On the other hand, it is reported that HA alone, i.e., Matrigel^®^ is overexpressed in breast cancer, providing increased response, and influencing the polarity of macrophages in many types of tumors [166]. To overcome that problem, many HA-composite 3D cell culture models have been formed. For instance, Baker et al. [160] formed an HA derivative crosslinked with matrix metalloproteinase (MMP)/oxime (HA-MMPx) modified with collagen and laminin. Such hydrogel composition allowed made it possible to obtain a 3D cell culture platform that was stable in physiological temperature, adequate mechanically, without distorted macrophage polarization and excessive response. The addition of laminin increased the hydrogel’s stress relaxation, enabling the native moves of MCF-10A mammary epithelial cells. This property of HA-MMPx significantly increased healthy tissue regeneration in comparison to Matrigel^®^. Moreover, the well-defined composition of HA-MMPx provided the highest invasion of immune cells, i.e., host natural killer (NK) cell infiltration in comparison to other cell culture platforms. The increased NK infiltration in breast cancer correlates with a better prognosis for patients. Thus, more advanced studies on this HA-MMPx platform could be a breakthrough in future anticancer therapies’ design.

Natural hydrogels seem to be perfect 3D tumor culture models; however, their mechanical properties range 0.1–0.4 kPa, which is insufficient from the perspective of tumors, whose mechanical properties depending on tumor type range ca. 1–6 kPa [180,181,182]. To overcome these problems, natural hydrogels are usually reinforced with other hydrogels showing increased mechanical properties or various types of nanomaterials.

Cellulose-based materials can help increase the mechanical properties of natural hydrogels. They can serve as hydrogel additives or hydrogels, and in the latest times, these materials have gained a lot of interest among both engineers and biologists [183,184,185,186]. For instance, collagen–nanocellulose hydrogel fairly mimics the ECM of pancreatic ductal adenocarcinoma (PDAC) [186]. The hydrogel consisted of short nanocrystals or elongated nanofibers (so-called nanocellulose) and cell adhesive proteins that provided adequate cell–materials surface interactions. Such composition not only provided stiffness in the range of 0.6–1.2 kPa that might be easily adjusted to mimic the lower profile of PDAC’s ECM but also decent proliferation and morphology of MIA PaCa-2 and PANC-1 tumor cells in 3D culture over 14 days.

In other studies, Shokri et al. [187] combined methylcellulose (MC), HA, and silk fibroin (SF) to obtain a thermosensitive physically crosslinked hydrogel platform (MCHASF) that mimics the native breast tumor ECM. The obtained model showed 6 weeks of physiological stability, similar mechanical properties (elastic modulus of ca. 1 kPa) to native breast tumor (1–4 kPa), and provided an environment that allowed the mimicking of the morphology of human breast cancer (MDA-MB-231) cells characteristic for native malignancy. This model also kept the native drug resistance and metastatic potential of MDA-MB-231. Additionally, the migration rate and overexpression of MMP2, MMP9, and VEGF proteins were increased, which proves the metastatic potential of the tumor that resembles native in vivo conditions.

#### 3.2.2. Synthetic Hydrogels

Although synthetic hydrogels make it possible to control many of the hydrogel parameters such as crosslinking rate or mechanical properties, hydrogels consisting of synthetic hydrogels are biochemically inert. To overcome that limitation, they can be modified with bioactive macromolecules or natural polymers [29,176,183].

Hybrid hydrogels are also beneficial approaches that serve as 3D cell culture models. Such combinations allow for overcoming the limitations of both, natural hydrogels, i.e., poor mechanical properties, and stability in physiological conditions, and synthetic hydrogels, i.e., biochemical inertia.

One of the examples of synthetic/natural hydrogel composites is gelatin methacrylate (GelMA) [9]. Shah et al. studied the GelMA 3D model which was seeded with patient neurospheres and U251 glioblastoma tumor cells. In this study, GelMa stiffeners ranged from ca. 5–19 kPa, which corresponds to the stiffness that is provided in natural glioblastomas environment, i.e., ca., 1–13 kPa. The results showed that 3D models provided a 3.5-fold increased invasive potential of U251 cells than those seeded on 2D models.

The 3D hydrogel models for tumor cell culturing are interesting approaches that allow a thorough investigation of the physiology of tumors, i.e., cellular morphology, phenotype, gene expression pattern, tumor–immune cell interactions, and drug resistance. Nevertheless, it should be noted that animal models based on rodents still are one of the best approaches to imitating tumor physiology. On the other hand, it should be noted that the preclinical testing results of drug efficacy on animal models rarely correspond to human clinical trials [167,188]. This is a result of differences in animal and human size, cells, and genes. Additionally, current ethical issues limit animal and human preclinical and clinical trials. Given the current state of knowledge, 3D cell culture models and animal models should be combined and used in preclinical trials. Although 3D cell culture models are promising approaches, at this time they will not replace small animal culture models. That is why there is still a huge demand for studies on designing and validating 3D cell culture models to provide an adequate physiologically, chemically, and mechanically complete environment for tumor testing that will reflect the physiology of the human organism [160].

## 4. Conclusions and Future Directions in Designing Biomaterials and 3D Cell Culture Models in Cancer Therapy

Anticancer therapies are a very challenging and complex field with many obstacles to overcome [189]. Many methods of forming biomaterials dedicated to anticancer therapies are being widely investigated [190]. The variations of materials manufacturing techniques could be observed, including co-axial electrospinning, emulsion electrospinning, bio-electrospraying, solution blow spinning, and various types of 3D printing and bioprinting. Scientists try to manipulate the parameters of production methods to receive a high-quality biomaterial with appropriate properties for anticancer therapy and regeneration of organs or tissues. Currently, most of these materials and methods are in the preclinical stage, although it is clear that their clinical applications are not far off [191].

The current trends in anticancer therapies are focused on combining biomaterials with chemotherapies, radiotherapies, and PTT to provide a synergistic effect that will result in winning the battle against cancer, promoting tissue regeneration, and preventing cancer relapse. Such an approach might help to face most of the anticancer therapies’ requirements, but it should also be remembered that the fabrication and implementation of such biomaterial is challenging and involves many laborious functionalizations and optimizations. It should be noted that biological systems are dynamic, and under NIR, properties such as stiffness can change, and in the effect can influence cell adhesion.

Another approach that could help in anticancer therapies is 3D cell culturing models. The idea behind using such approaches is a thorough investigation of tumor physiology. Many scientists are working on a new type of 3D cell culture model [192]. Despite 3D cell culture models offering many advantages such as the examination and understanding of gene expression patterns, tumor–immune cell interactions, and tumor resistance to anticancer drugs, there is still plenty to do in this field. However, the future perspectives of 3D cell culture models and scaffolds assume their functionalization by incorporating endothelial and stromal cells to induce the vascularization process. Such an approach will be more functional and approximate these materials to the animal models [167].

There are still many problems to resolve in the modern anticancer drug delivery systems. In the field of drug delivery systems there is a need for improvements in targeted and controlled rate and dose of drug release in a desired localization, for example, in the vicinity of the cancer tumor. Numerous research teams [193] are still working on more realistic solutions. The long-term goal is a high-standard drug delivery and release system. Very small but targeted doses in targeted therapy can change patients’ quality of life. This can eliminate side effects and increase tissues’ and whole organs’ regenerative capacity.

To summarize, many proposals and applications of electrospun nanofibers, 3D-printed materials, and hydrogels discussed in this paper concern the stage of in vivo tests or proof-of-principle studies. Scientists from various universities are trying to translate these ideas and accomplishments into clinical trials. The long-term goal is to overcome the current problems of implants and transplants, drug delivery, and cell culture strategies, resulting in designing more successful cancer therapies.

## Figures and Tables

**Table 1:** Overview of degradable and non-degradable polymers and their biomedical applications in cancer treatment and regenerative medicine.

Polymer	Method of Fabrication/Type of Material	Application	Ref.
Polycaprolactone (PCL)	Electrospinning	Skin tissue regeneration, wound healing, skin cancer	[91]
Polyethylenoxide (PEG)	Electrospinning	Tissue regeneration, especially soft tissue regeneration	[92]
Polycaprolactone (PCL) -gelatin	Electrospinning	Tracheal tissue engineering applications	[93]
Poly(l-lactide-co-glycolide) (PLGA)	Electrospinning	Neural tissue engineering applications (axons stretching)	[94]
Poly(lactic-co-glycolicacid) (PLGA)	Electrospinning	Skeletal muscle regeneration after muscle tumor	[95]
Polymeric/solid/hydrogels	Nanoparticles, microparticles	Ocular drug delivery, eye cancer, implants	[96]
Polycaprolactone- Polydimethyl Siloxane (PCL-PDMS)	Electrospinning	Bone tissue engineering, bone cancer	[97]
Polycaprolactone- Polylactic acid PCL/PLA	Coaxial Electrospinning	Bone tissue engineering, bone cancer	[98]
Polycaprolactone- poly(l-lactide-co-glycolide) PCL/PLGA	3D printing/Electrospinning	Bone tissue engineering, bone cancer	[99]
Polylactic acid (PLA)	Electrospinning	Tendon/joint stability	[100]
Polylactic acid (PLA)	Electrospinning	Soft tissue engineering/repair and regeneration of tendon defects and injuries	[101]
Polycaprolactone (PCL)	Electrospinning	Vascular graft	[102]
Poly(vinylidene difluoride-Trifluoroethylene) (PVDF-TrFE)	Electrospinning	Neural tissue engineering/regenerative medicine	[103]
Poly(glycerol sebacate)	Electrospinning	Tissue engineering/soft tissue	[104]
PLGA/collagen		Skin	[105]
Gelatin/cellulose acetate/elastin	Electrospinning	Skin/biomimicking	[106]
Laminin-functionalized PDLLA	Electrospinning	Skin cancer/skin healing	[107]

**Table 2:** Electrospun scaffolds combined with PTT effect.

Material	Multifunctional Effect	Light WaveLength	Application	Ref.
PLA/PCL with Cu_2_S	- high mortality (>90%) of skin tumor cells - inhibited tumor growth	808 nm	Skin cancer therapy and wound healing	[111]
PCL microfiber/GO scaffold	- cancer cell in situ (≈98%) removal	810 nm	Breast cancer therapy/adipose tissue repair	[112]
PCL/Gelatin scaffold with BP nanosheets	- create a tumor-suppressive microenvironment - increased tissue repair ability	808 nm	Skin cancer treatments	[113]
Gelatin/Au	- PTT effect supports cell adhesion and proliferation	805 nm	Breast tumor cells	[114]
PLA/PCL composite scaffolds	- inhibit in vivo tumor growth - advance healing of cancer-surgery caused wounds	808 nm	Skin cancer cells	[115]
PCL/Gelatin	- control of drug release by PTT effect	808 nm	Inhibition of cancer tumor growth	[116]
PCL	- Control of drug release	808 nm	- chemotherapy with PTT effect	[117]

**Table 3:** The three-dimensional printing methods as a promising tool in anticancer therapies and PM.

Method	Advantages	Disadvantages	Ref.
Stereolithography: photopolymerization of liquid material	- High quality of printed objects - Low thermal stress	- Cytotoxicity of the products - Post-curing processes - Material must have a photo-curable properties	[125]
Selective laser sintering (SLS): laser energy absorbing material (powder)	- Solvent-free and fast printing - high quality	- High energy may cause anticancer drug degradation - Limited number of materials with laser absorbing properties	[126]
Inkjet 3D printing: ink (drug solution) and substrate (polymer-based material)	- Continuous printing prevents clogging of the needle - Low cost - High precision	- Low resolution - High cost of production	[127]
Fused deposition modelling (FDM): printing using filaments	- Low cost - No post-production - Solvent-free process	- Only thermoplastic polymers - Preparation of filaments - Heat can cause the degradation	[128]

**Table 4:** Three-dimensional printed scaffolds combined with PTT effect.

Material	Multifunctional Effect	Light Wavelength	Application	Ref.
carbon-embedding larnite (larnite/C) /CaCO_3_	- destroying human osteosarcoma cells (MNNG/HOS) - increased bone regeneration ability PTT effect	808 nm	Bone cancer treatment	[148]
Niobium carbide (Nb_2_C) MXene-modified 3D-printing biodegradable bioglass (BG@NbSiR)	- increased bone regeneration ability - providing long-term immune therapy PTT effect	808 nm	Breast cancer/bone metastasis therapy	[149]
Fe-CaSiO_3_	- excellent mechanical properties - ROS production - increased bone regeneration ability PTT effect	808 nm	Bone cancer treatments	[150]
SrCuSi_4_O_10_/PCL	- increased bone regeneration ability - enhanced vascularized bone regeneration PTT effect	650–1000 nm	Bone cancer treatments (osteosarcoma, chondrosarcoma, fibrosarcoma, etc.)	[151]
MoS_2_/PLGA/BG		808 nm	Bone cancer treatments	[152]

## Data Availability

Not applicable.
